# Heat shock proteins in multiple myeloma

**DOI:** 10.18632/oncotarget.1584

**Published:** 2014-01-19

**Authors:** Lei Zhang, Jacqueline H.L. Fok, Faith E. Davies

**Affiliations:** ^1^ Haemato-Oncology Research Unit, Division of Molecular Pathology, Cancer Therapeutics and Clinical Studies, The Institute of Cancer Research, London, UK

**Keywords:** heat shock proteins, multiple myeloma, protein folding, ER stress, haematology

## Abstract

Heat shock proteins are molecular chaperones with a central role in protein folding and cellular protein homeostasis. They also play major roles in the development of cancer and in recent years have emerged as promising therapeutic targets. In this review, we discuss the known molecular mechanisms of various heat shock protein families and their involvement in cancer and in particular, multiple myeloma. In addition, we address the current progress and challenges in pharmacologically targeting these proteins as anti-cancer therapeutic strategies

## INTRODUCTION

Heat shock proteins are a group of highly conserved proteins found in both eukaryotic and prokaryotic cells. They are involved in a wide range of cellular processes such as assisting protein folding and degradation of misfolded proteins, intracellular trafficking, modulating signalling pathways and regulating immune responses [1-5]. The multi-functional nature of heat shock proteins enables them to play critical roles in the regulation of protein homeostasis and cell survival. Although the proteins are frequently associated with the cellular stress response, they also play an important role in supporting normal cellular processes such as development and differentiation [6-8].

They were accidentally discovered in 1962, as a set of genes whose expression is elevated by heat shock in *Drosophila melanogaster* [9]. It is now known that heat shock proteins function as molecular chaperones and can play many roles in the cell in addition to modulating the heat shock response. In mammalian cells, they are classified into five families according to their molecular weight: Hsp100, Hsp90, Hsp70, Hsp60 and small heat shock proteins including Hsp27. Members of each family can be either constitutively expressed or cell event induced, and can be found in defined cellular compartments carrying out specific functions.

There are numerous lines of evidence which link heat shock proteins to the pathogenesis of cancer. They are found to be overexpressed in a wide range of cancers and are implicated in cell survival, apoptosis, invasion, metastasis and escape of immune surveillance. As a tumour progresses, it becomes increasingly dependent on these proteins to adapt to its microenvironment and to stabilise the large amount of oncogenic proteins produced which support growth and survival. The different heat shock protein families are being studied extensively as potential anti-cancer targets for two main reasons: (1). heat shock proteins interact with multiple cancer related client proteins/pathways and targeting them may lead to the inhibition of multiple cancer causing pathways; (2). some cancers rely on heat shock proteins to survive the proteotoxic stress induced by the production of excessive proteins/oncogene products.

Multiple myeloma is a cancer resulting from the malignant proliferation of plasma cells in the bone marrow and one important feature of myeloma plasma cells is the secretion of excessive monoclonal paraproteins [10]. Despite recent advances in treatment and the use of high dose chemotherapy, the majority of patients relapse even after successful initial treatment. To date, the disease remains incurable with a median survival of 4 years. There is therefore an urgent need for better treatments and new drugs. In recent years, heat shock proteins have become attractive potential therapeutic targets in multiple myeloma, as the ability to deal with proteotoxic stress as a result of paraprotein production is critical for myeloma cell survival [10, 11]. Importantly, several inhibitors of Hsp90 have demonstrated activity against myeloma cells *in vitro* and *in vivo*, and clinical trials are ongoing [12-15].

In this review, we aim to provide an overview of the known mechanism and functions of the various heat shock protein families and their implication in cancer development and progression concentrating particularly, on multiple myeloma. We also discuss the role of heat shock proteins as potential therapeutic targets in multiple myeloma and discuss the supporting pre-clinical and clinical data.

### The mechanisms and functions of heat shock family proteins

### Hsp90

Multiple Hsp90 family proteins exist in different subcellular locations. These include the cytoplasmic Hsp90α (inducible) and Hsp90β (constitutive), mitochondrial TNF receptor-associated protein 1 (TRAP1) and endoplasmic glucose regulated protein 94 (Grp94). All of the proteins are highly abundant and the cytoplasmic isoform is essential for cell survival [16], and studies in yeast demonstrate that Hsp90 may interact with more than 10% of the yeast proteome [17]. Unlike other heat shock proteins involved in general protein folding tasks, Hsp90 is found to interact only with a group of selective client proteins, many in a more mature folding conformation compared to Hsp70 substrates. Rather than protein folding, Hsp90 is more commonly associated with client protein maturation or functions to maintain a client protein in a specific folding conformation required for its activity, for example, to respond to activation signals such as phosphorylation. The client proteins identified to date consist mainly of protein kinases, receptors and transcription factors, and many of these are involved in cell cycle control and signalling pathways [18-24]. The list of client proteins identified for Hsp90 is increasing, yet the molecular basis for substrate selectivity is still largely unknown as client proteins have no obvious common sequence motifs. A current list of Hsp90-interacting proteins has been maintained by Didier Picard and can be found at (http://www.picard.ch/downloads/downloads.htm), which includes client proteins as well as co-chaperones.

**Figure 1:**
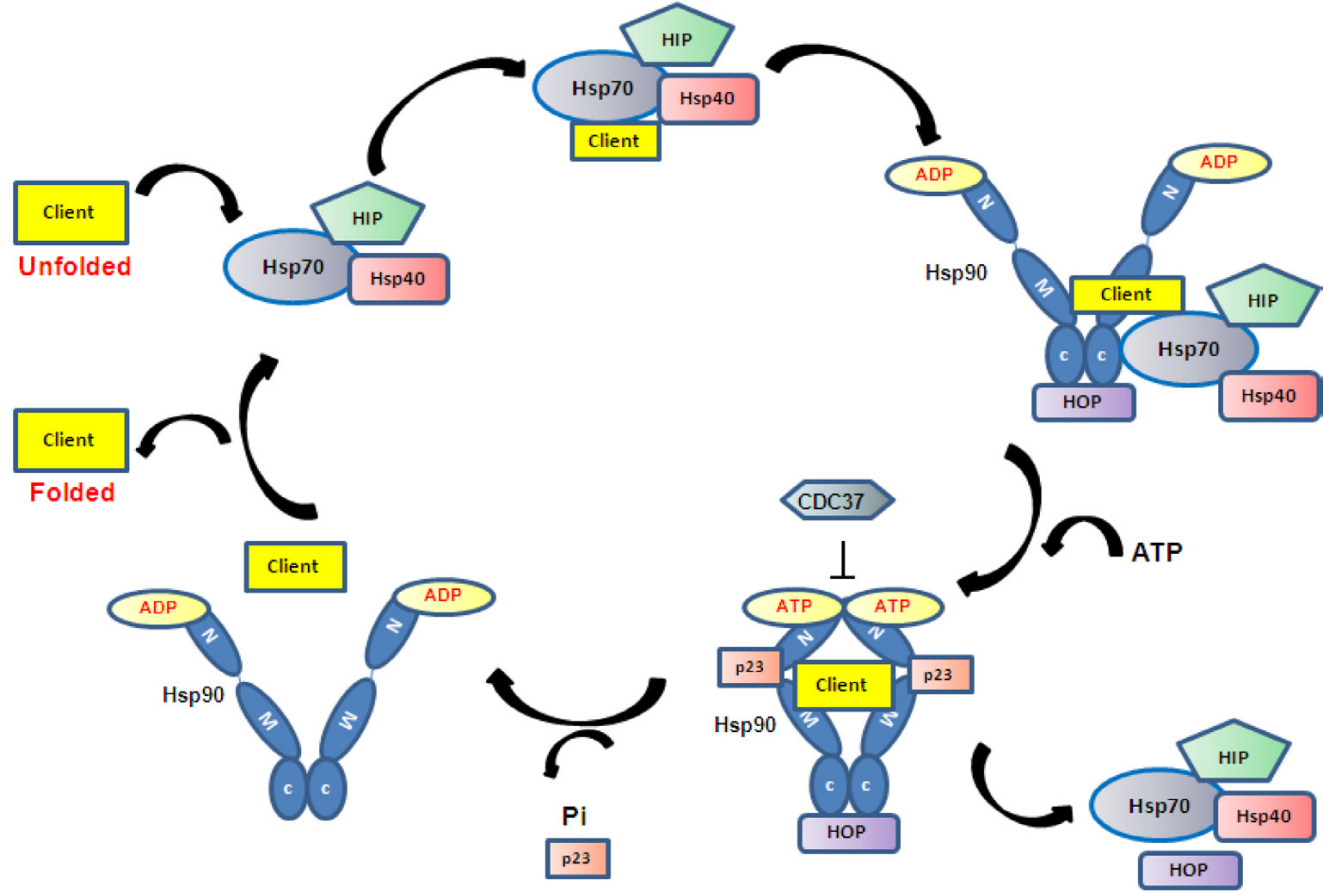
The Hsp90 chaperoning system ATP binding and hydrolysis drive Hsp90 conformational changes resulting in the binding and release of client proteins. Client proteins are presented to Hsp90 by the Hsp70 chaperone complex.

Hsp90 proteins exist as homodimers of subunits consisting of an N-terminal ATPase domain, a C-terminal dimerisation / protein interaction domain, and a middle domain associated with client protein binding [25]. ATPase activity is essential for the chaperoning activity of Hsp90 [26, 27]. Following the addition of ATP, Hsp90 undergoes a conformational change, which induces an open to shut conformation shift [28], with transient dimerisation of the N-terminal domains and N-M domain association (Figure 1) [29, 30]. The middle segment of Hsp90 has been identified as the binding site for protein kinase PKB/Akt and is implicated as the main site for client protein interactions [31]. This segment can also interact with cochaperones and is required for N-terminal ATPase activity. The C-terminal domain is involved in dimerisation and contains a highly conserved EEVD sequence which is required for the binding of tetratricopeptide repeat (TPR) containing family of cofactors, such as HOP [32, 33]. The C-terminal domain also contains an alternative ATP-binding site [34, 35], but how this contributes to the overall function of Hsp90 remains unclear.

The Hsp90 homodimer functions like a molecular clamp, using ATP binding and hydrolysis to drive the confirmation change cycle of Hsp90 which is required to facilitate the binding and release of client proteins. It works in a multi-chaperone complex and the current proposed mechanism is that client proteins first bind to Hsp70. ATP hydrolysis of Hsp70 by Hsp40 stabilises the initial client/Hsp70/40/HIP complex, which then interacts with Hsp90 in ADP-bound open conformation via Hop, presenting the client protein to Hsp90 [36]. When Hsp90 exchanges ADP to ATP, its open to shut conformational change leads to the dissociation of Hsp70/40 and HOP and the association of another set of co-chaperones such as CDC37 and p23 to form the mature complex [37, 38]. In this mature state the client protein becomes activated (Figure 1). Studies of glucocorticoid receptor (GR) activation demonstrate that Hsp90 and Hsp70 are absolutely required for GR activation (i.e. opening of the steroid binding cleft in GR), whereas cochaperones such as Hop, Hsp40 and p23 help to facilitate the chaperoning activity by client protein recruitment (HOP), ATP hydrolysis of Hsp70 (Hsp40) and stabilisation of Hsp90-ATP conformation (p23) [39-41]. To date more than 20 Hsp90 co-chaperones have been identified and all are involved in the recruitment of client proteins, control of client protein maturation and modulation of ATPase activity. It is thought that the binding and release of specific co-chaperones in an orderly way may control the activity / selectivity of Hsp90, and that different client proteins require a different set of co-chaperones. Given the complexity of the Hsp90 chaperoning system, a full understanding of the molecular mechanism is still lacking.

In addition to the cytoplasmic Hsp90s, protein quality control requires the functions of compartment specific Hsp90s located within various cellular organelles. TRAP1 is an Hsp90 located in the mitochondria involved in mitochondrial protein folding, cytoprotection and mitochondrial integrity [42, 43]; whereas Grp94 is the only Hsp90 residing in the endoplasmic reticulum (ER), where it plays a critical role in regulating ER protein homeostasis by chaperoning highly selective client proteins such as immunoglobulins [44], targeting misfolded client proteins for ER-associated degradation (ERAD) [45] and storing Ca^2+^ to regulate ER calcium flux [46]. Both are implicated in promoting tumour progression [43, 46].

### Hsp70

Eukaryotic cells also express a range of Hsp70 proteins in various subcellular localisations. Family members include the constitutively expressed Hsc70 and stress induced Hsp72 in the cytoplasm, Bip (Grp78) localised in the endoplasmic reticulum and mortalin/Grp75 in the mitochondria. Similar to Hsp90, Hsp70 protein consists of a N-terminal ATPase domain where ATP exchange acts as the driving force of the conformational change required for target protein binding and release; a substrate binding domain with affinity for hydrophobic amino acid residues; and a C-terminal domain containing an EEVD motif for co-chaperone binding and functioning as a ‘lid’ which controls the availability of the substrate binding domain to target proteins [47]. Hsp70 forms a complex with its cochaperone Hsp40 and a nucleotide exchange factor such as Bag-1 and HspBP1. Hsp40 stimulates Hsp70 assisted protein folding by interacting with Hsp70 and promoting ATP hydrolysis, resulting in a closed conformation and tight binding of substrate, whereas a nucleotide exchange factor stimulates the release of ADP and binding of ATP, thereby opening the binding pocket for substrate release [48].

During protein synthesis, partially synthesized and incompletely folded polypeptide chains expose hydrophobic regions that need to be protected from misfolding and aggregation. Hsp70 assists the *de novo* folding of 15-20% of all bacterial proteins, and this figure is thought to be even higher in eukaryotes [3]. It interacts with a wide spectrum of nascent polypeptide chains co- and posttranslationally, with preference for chains between 30-75kDa [49-51]. It utilises ATP driven cycles of substrate binding and release to carry out chaperoning functions, preventing aggregation by maintaining a low free substrate concentration, while enabling free substrate to fold to its native state [52-56]. On the other hand, the binding and release cycles may also induce specific unfolding of a misfolded polypeptide or pull apart aggregated proteins for them to be refolded to their correct state [56].

**Figure 2:**
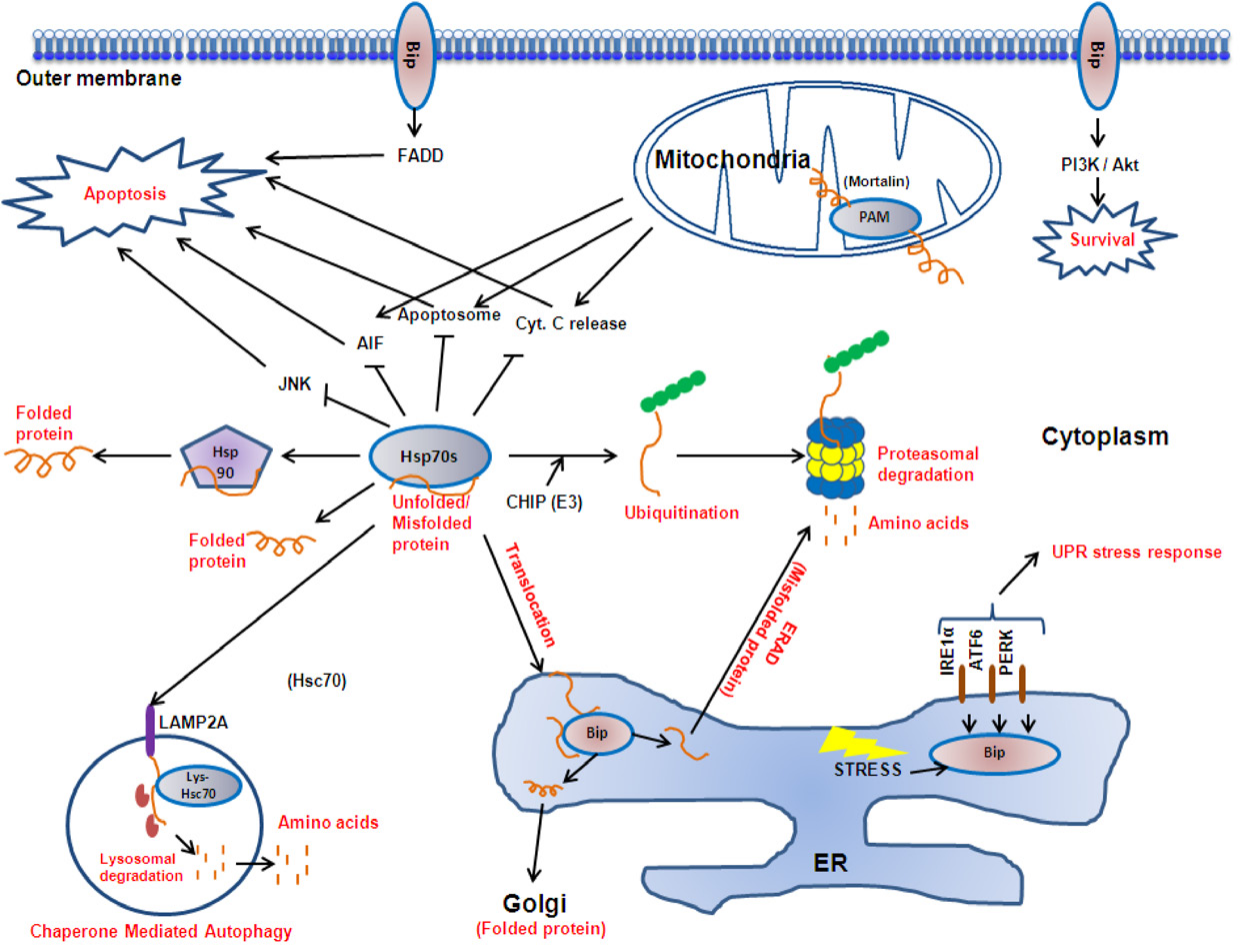
The Hsp70 family proteins Hsp70 protein isoforms (Bip, cytoplasmic Hsp70s, lys-Hsc70 and mortalin) reside at various subcellular localisations to perform specific roles in protein folding, translocation, degradation and signal transduction, thereby mediating cell survival and apoptosis.

The family members found at different cellular localisations fulfill specific roles. Collectively, they form a key part in the cellular mechanism maintaining protein homeostasis and cell survival (Figure 2). They play central housekeeping functions in the cell as part of a complex network working with co-chaperones and downstream chaperoning systems such as Hsp90. In addition to assisting the folding of newly synthesized and refolding of misfolded proteins discussed above, they translocate target proteins across membranes [57], as well as directing protein degradation by the ubiquitin-proteasome pathway [58] or autophagy [59]. An increasing number of signal transduction proteins and transcription factors are known to transiently interact with the Hsp70 complex [60], and together with the Hsp90 complex, the Hsp70 system is linked to cell cycle regulation, apoptosis and differentiation.

The endoplasmic reticulum resident Bip is involved in the folding and assembly of proteins in the ER, targeting misfolded proteins to ER-associated protein degradation (ERAD) and signaling the unfolded protein response in response to stress [61-63]. Bip may also be expressed on the cell surface of some tissues and is involved in signal transductions [64-66]. Mitochondria matrix localised Hsp70 (mortalin) forms part of the presequence translocase-associated motor (PAM) complex which acts as the driving motor of protein translocation from the cytoplasm into the mitochondria [67]. Whereas cytosolic Hsp70 is required for the post-translational translocation of secretory proteins destined to the ER, by holding the fully transcribed polypeptide in an incompletely folded state for translocation [68, 69]. In addition the cytoplasmic inducible Hsp72 and its cognate protein Hsc70 are responsible for the folding of proteins in the cytoplasm as well as the recruitment of E3 ubiquitin ligases such as CHIP to tag target proteins for proteasomal degradation [70]. Hsc70 also participates in chaperone-mediated autophagy, a type of lysosomal degradation which selectively targets specific proteins. Cytosolic Hsc70 binds to a target protein and presents it to the lysosome receptor LAMP-2A. At this site the substrate protein is subsequently unfolded and translocated into lysosome, a process which is assisted by the lysosomal resident Hsc70 [71, 72]. In addition to their roles in maintaining the cellular protein program, cytoplasmic Hsp70 inhibits both the caspase dependent and independent apoptosis pathways at multiple levels [73, 74].

### Small heat shock proteins - Hsp27

In contrast to Hsp90 and Hsp70, small heat shock proteins are a family of ATP-independent chaperones. With a small size between 15-30kDa, they oligomerise to form homo or hetero-oligomers with up to 50 subunits [75], which determines their chaperoning activity. In addition to their phosphorylation status, cell-cell signalling and various protein modifications also modulate their oligomerisation [76].

Hsp27 belongs to this family and functions to prevent protein aggregation by directly binding misfolded substrates, and promoting protein refolding by interaction with the Hsp70 chaperone complex. In addition, Hsp27 can directly prevent cell death by interfering with key components of the apoptosis pathway, such as blocking the formation of the apoptosome by binding to cytochrome c released from the mitochondria [77], and by interacting with Daxx, a mediator of Fas-induced apoptosis [78].

Under stress conditions, Hsp27 is also directly involved in the ubiquitin-proteasome pathway by binding to the 26S proteasome and multi-ubiquitin chains, to facilitate the degradation of a selective range of target proteins [79]. By doing so, Hsp27 can mediate its cytoprotective effect at multiple levels by facilitating the degradation of various apoptotic and cell cycle proteins. For example, Hsp27 can enhance the anti-apoptotic activtity of the transcription factor NF-κB, as the presence of Hsp27 in the proteasome-protein substrate complex is required for the degradation of I-κBα, the inhibitor of NF-κB [79]. Hsp27 can also promote the degradation of the cell cycle inhibitor p27^Kip1^, thereby avoiding cell cycle arrest during stress [80].

### Hsp60/Hsp10

Hsp60 and Hsp10 form the mitochondrial chaperonin complex, which is involved in mitochondrial protein folding. The understanding of the structure and function of chaperonin has mainly come from studies performed on the bacterial chaperonin, GroEL and GroES.

GroEL (Hsp60) is an oligomer formed by monomers arranged into two stacked heptameric rings [81, 82], resulting in a barrel like cavity where misfolded or unfolded substrate proteins are folded. GroES (Hsp10), which forms a single heptameric ring, acts as a lid to the chamber and can bind to either end of the double GroEL rings [83, 84]. Like Hsp70 and Hsp90, ATP cycles induce conformational changes required for substrate protein folding. ATP and polypeptide binds to one GroEL ring, followed by GroES capping, resulting in the encapsulation of polypeptide in a hydrophilic cavity which promotes protein folding conditions [84]. Once the substrate is inside the chamber, ATP is hydrolysed slowly, allowing time for the protein to fold. The two rings of GroEL act in an alternate fashion [85], with ATP hydrolysis in one ring resulting in a structural transition in the opposite ring making it available for ATP binding, which in turn triggers the release of GroES and substrate protein from the original ring. A substrate protein may go through multiple binding and release cycles to reach its folded state [85].

In Eukaryotes, Hsp60 was first shown to reside in the mitochondria, and following interaction with Hsp10, is responsible for chaperoning nascent polypeptides as well as transporting target proteins from the cytoplasm into the mitochondria [86, 87]. Evidence also suggests that Hsp60 participates in apoptosis by interactions with mortalin (mitochondrial hsp70), p53 and survivin [88-90]. Accumulating evidence suggests that Hsp60 is not just a mitochondrial protein, as it also resides in the cytoplasm and unlike other heat shock proteins that mostly have pro-survival functions, Hsp60 has either pro-survival or pro-apoptosis functions [91]. It has also been found on the cell surface where it is involved in the activation of immune system [92, 93] and in the extracellular matrix where it has pro-inflammatory functions [94, 95]. The molecular mechanism of Hsp60 in humans remains largely unknown, but its involvement in cancer as well as its potential applications in cancer therapy is actively being investigated.

### Hsp110 (Hsp105)

Hsp110/105 is abundant in the cytosol of mammalian cells but relatively little is known about its function compared to the other heat shock proteins. It has diverged from the Hsp70 superfamily, and has independent chaperone activity as well as serving as a nuclear exchange factor to Hsp70s [96]. As an independent chaperone, unlike Hsp70, Hsp110 cannot assist protein folding, but acts to prevent protein aggregation of denatured proteins with higher efficiency compared to Hsp70 [97, 98]. It also exhibits differential substrate binding properties to Hsp70s with preference for substrates with aromatic residues, and this may account for the different chaperone activities of Hsp110 and Hsp70 [99].

### Hsf1

It is widely established that Hsf1 is the “master regulator” of heat shock protein expression. In the absence of stress, inactive Hsf1 monomers are held in a complex with Hsp70/Hsp90. At the onset of proteotoxic conditions, Hsf1 is released from the complex, homo-trimerises, translocates to the nucleus and activate the transcription of its downstream targets by binding to the heat shock elements in the promoter regions of target proteins [100, 101]. Hsf1 is classically recognised as a regulator of heat shock protein expression with its downstream targets such as Hsp72 and Hsp27. Several recent genome-wide analysis using ChIP and microarray technologies along with Hsf1 siRNA in yeast [102] and mammalian cells [103, 104] have uncovered a plethora of previously undiscovered Hsf1 gene targets, including genes implicated in transcriptional, RNA splicing, ubiquitylation, stress defence, vesicular transport and cell structures. Page *et al* [103] emphasized that aside from the chaperones induced by Hsf1 upon heat shock, the second most substantially induced group were genes coding for anti-apoptotic proteins. These genome-wide analyses also reveal the role of Hsf1 in regulation of stress, cellular adaptation, survival, development and disease.

### Heat shock proteins contribute to cancer progression and metastasis

Cancer cells proliferate at a fast rate and in order to survive, they resist apoptosis, upregulate oncogenes/oncoproteins, cope with environmental stresses such as hypoxia, and modulate various survival signalling pathways. Therefore, in order to overcome the challenging hostile environment, cancer cells have higher metabolic requirements for chaperones than non-cancer cells.

### Hsp90

A large number of oncoproteins, including cell cycle proteins, tyrosine kinases, signalling transduction proteins, anti-apoptotic proteins and transcription factors are known Hsp90 client proteins. Upregulation of Hsp90 has been widely observed in a range of solid and haematological malignancies including myeloma [105-109] and is required for the stability and function of these oncoproteins thereby supporting tumour development and survival. By supporting the large number of client proteins involved in multiple cancer related pathways, Hsp90 is involved in the regulation of many of the “hallmarks of cancer”, namely sustaining proliferative signalling, resisting cell death, evading growth suppressors, inducing angiogenesis, enabling replicative immortality, invasion and metastasis, and emerging hallmarks including deregulating cellular energetic and avoiding immune destruction [110, 111]. In addition, high expression of Hsp90 is an independent prognostic marker in a number of cancers. In breast cancer, it is associated with decreased survival [109], and in gastric cancer, high Hsp90 expression is linked to poor prognosis and tumour aggressiveness [112]. In CML, Hsp90 correlates with disease state and high levels are associated with resistance to therapy [113].

### Hsp70

Unlike Hsp90 which chaperones specific ‘client proteins’, Hsp70 family proteins assist general folding of unfolded or misfolded proteins exposing hydrophobic regions and prevent their aggregation. High Hsp70 expression is correlated with poor prognosis in a wide range of cancers such as breast, endometrial, cervical, oral and bladder carcinomas and has been extensively reviewed elsewhere [114]. The anti-apoptotic role has also linked Hsp70 to chemotherapeutic resistance in ovarian cancer and leukaemia [115, 116]. Hsp70 is involved in multiple cancer promoting pathways by associating with the Hsp90 chaperone system as well as carrying out independent functions in apoptosis, senescence, and protein regulatory pathways such as autophagy [117].

The cytoplasmic Hsp70s regulate the apoptosis pathway at multiple levels, for example, Hsp70s have been shown to protect Bcl-2 from proteasomal degradation [118]; block Bax translocation to the mitochondria thereby preventing cytochrome c release [119]; bind Apaf-1 and prevent the recruitment of caspase-9 to the apoptosome [120, 121]; and to prevent AIF translocation to the nucleus to cause chromatin condensation and DNA degradation [122, 123]. It is interesting to note that the function of Hsp70s do not always rely on their ATPase activity, for instance it has been shown that Hsp72 inhibits JNK activation independently of its chaperoning activity [124-126]. The Hsp70s also play a protective role against senescence. Hsp72 knock down induces senescence in a variety of cancer cell lines [127, 128] and Hsp72 controls Her-2-induced senescence by regulating p21 and survivin in a mouse breast tumour model [129]. Evidence also suggests that Hsp70 supports autophagy by maintaining protein homeostasis and supporting cancer cell survival. Hsp70 localises at the autophagosome/ lysosomal membrane compartments and inhibits lysosomal permeabilisation [130, 131]. In addition, Hsp70 participates in chaperone mediated autophagy by delivering target proteins to the lysosome surface receptor LAMP-2A, where it enables their translocation into the lysosomal lumen (Figure 2) [71, 132].

### Small heat shock proteins - Hsp27

Hsp27 is also commonly overexpressed, correlating with prognosis and chemoresistance in many cancers including colorectal [133], breast [134], prostate [135] and ovarian [136]. Elevated expression is associated with tumour aggressiveness in both primary and metastatic tumours. Apart from having anti-apoptotic roles at multiple levels contributing to primary tumour survival, Hsp27 is involved in actin dynamics and is overexpressed in metastatic breast tumour contributing to cell migration and invasion. Silencing of Hsp27 leads to decreased bone metastasis in a breast tumour model [137]. In addition, Hsp27 is implicated in epithelial-to-mesenchymal transition (EMT) in breast [138], lung [139], and has been shown to be a key mediator of both IL-6 dependent and independent EMT in prostate cancer [140]. Experimental models also suggest that Hsp27 can promote angiogenesis by NFkB dependent upregulation of VEGF-gene transcription and secretion of VEGFR-2 in endothelial cells [141]. Knocking down Hsp27 in breast cancer cells reduced endothelial cell proliferation and reduced secretion of VEGF and FGF [142].

### Hsp60/Hsp10

Increasing evidences suggest that Hsp60 and Hsp10 may also be important players in cancer progression. As reviewed by Cappello *et al* [143], Hsp60 expression is altered in a wide range of cancers with potential diagnostic and prognostic implications. As well as assisting protein folding in association with Hsp10, cytosolic Hsp60 can regulate apoptosis by stabilizing the apoptosis inhibitor survivin [89] and binding to and inhibiting pro-apoptotic Bax and Bak [144]. Conversely, Hsp60 can also promote the activation of caspase-3, leading to tumour cell death [145]. Hsp60 interacts with β-catenin—a key oncogene driving cancer development and metastasis, where it is found to enhance β-catenin transcriptional activity thereby promoting metastasis [146]. Cell surface Hsp60 also directly interacts with and activates α3β1 integrin, which can contribute to tumour progression and metastasis [147].

Although Hsp10 is mostly considered to reside in the mitochondria as a component of the Hsp60/Hsp10 chaperonin complex, increasing evidence suggest that Hsp10 may have Hsp60-independent roles. This hypothesis is supported by the differential expression and localisation of these two heat shock proteins in tumour cells [148]. In tumour cells, Hsp10 is found to accumulate in the cytoplasm [148], and may be involved in the inhibition of apoptosis by altering the expression level of Bcl-2 family proteins [149]. Hsp10 may also contribute to tumour progression through its role in the regulation of the Ras GTP-ase signalling pathway [150]. In addition, Hsp10 can be released from tumour cells and evidence suggests that Hsp10 may enable tumour cells to escape immune surveillance by suppressing T cells expressing CD3 zeta chain, inhibiting cytokine production [151].

### Hsp110 (Hsp105)

Finally, Hsp110 may have a potential use in cancer as a marker of prognosis and drug response. It is overexpressed in malignant melanoma [152], colorectal [153] and pituitary tumours [154], and high expression is associated with advanced and metastatic lesions [153, 155]. In contrast, a reduction in Hsp110 expression was correlated with invasion and metastasis and therefore poor prognosis of oesophageal cancer [156]. A truncated mutant of Hsp110 has been found in colorectal cancer with microsatellite instability, and in this type of cancer the truncated mutant inhibits the protective role of the wild type form in a dominant negative manner. High expression of the truncated mutant is linked to chemo-sensitivity and better prognosis [157].

### Heat shock proteins are potential therapeutic targets in multiple myeloma

Multiple myeloma is characterised by the production of large quantities of nascent immunoglobulin [10]. As a result, myeloma cells rely on their protein handling mechanisms to cope with protein load and maintain survival. A number of pathways are responsible for protein homeostasis in the cell including the unfolded protein response (UPR), ubiquitin proteasome pathway, autophagy and aggresome pathway. The endoplasmic reticulum (ER) is a site of protein folding and quality control. The accumulation of unfolded/misfolded proteins in the lumen of the ER triggers the UPR, which activates downstream pathways to inhibit protein translation and increases protein folding capacity by upregulating molecular chaperones [158]. If proteins cannot be correctly folded in the ER, they are retrotranslocated to the cytoplasm to be ubiquitinated and destroyed by the proteasome [159]. Alternatively, excess proteins can be removed by autophagy via lysosomal degradation, which can be upregulated during stress triggered by protein aggregation, nutrient deprivation, or proteasome inhibition [160-164]. When protein folding and degradation capacity is exceeded, unfolded/misfolded protein aggregates in the cytoplasm are transported along the microtubule to the microtubule organising centre, where they form aggresomes [165, 166]. The aggresomes act as storage centres for toxic proteins until these proteins are eventually targeted to chaperones for refolding or degradation by autophagy [167, 168].

The protein handling pathway is a tightly linked process and is overloaded by the large amount of immunoglogulin produced in myeloma. As a result, the protein handling pathway is actively being explored as an attractive therapeutic target in myeloma. The success of the clinically approved proteasome inhibitor bortezomib provides evidence that targeting this pathway can be an effective treatment strategy in myeloma. Efforts have therefore been put into developing inhibitors of the UPR, heat shock proteins, proteasome, autophagy and aggresomes, and these have been extensively reviewed by Aronson *et al* [161].

**Figure 3:**
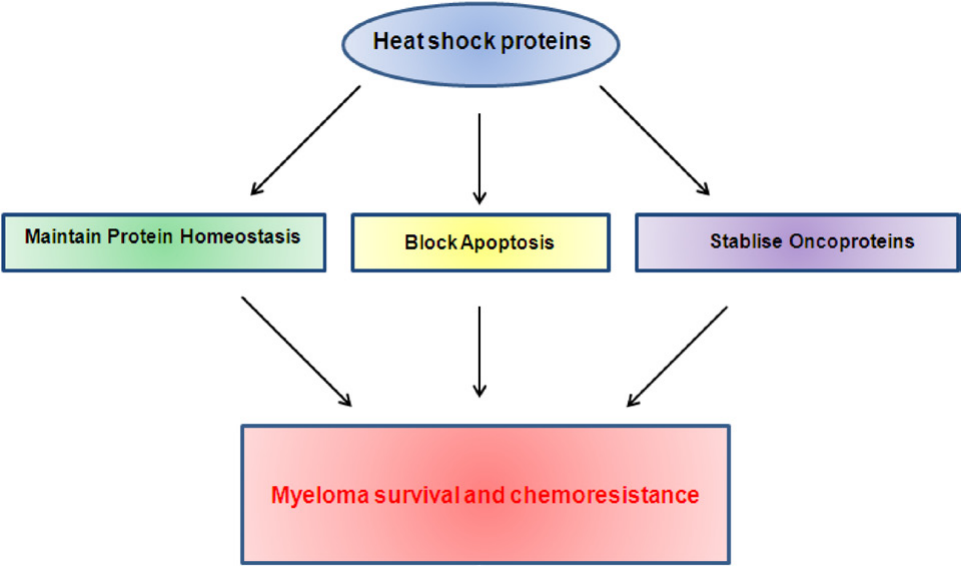
Heat shock proteins contribute to myeloma survival and chemoresistance via their roles in multiple pathways known to be important in myeloma

Being molecular chaperones responsible for protein folding, the heat shock proteins play a key role in all of the protein homeostasis pathways and thus the handling of immunoglobulin folding in myeloma. In addition to their chaperoning functions, heat shock proteins are found to be involved in many other signalling pathways important for myeloma growth and survival, making them particularly attractive targets (Figure 3).

Numerous studies have shown that Hsp90 inhibition in myeloma cells induces apoptosis and depletes multiple client proteins such as AKT, STAT3, IL-6Rα, thereby simultaneously disrupting multiple pathways known to contribute to cell survival, including the JAK/STAT, PI3K, NF-κβ, and RAS/ERK pathways [169]. Cytoplasmic Hsp90 has also been shown to be a modulator of the UPR by associating with and stabilising IRE1 and PERK, two major transactivators of the UPR responsible for the downstream upregulation of stress response genes and translational repression [170]. It is also shown that Hsp90 inhibition induces UPR in myeloma, and that Hsp90 inhibitors induce myeloma cell death at least in part via the UPR death pathway [11]. Given the support of myeloma cell growth and survival by the bone marrow microenvironment, the ability of HSP90 inhibition to overcome exogenous IL-4-induced chemotherapy resistance highlights the potential efficacy of HSP90 inhibitors *in vivo* [171].

Preclinical studies demonstrate that the inhibition of Hsp90 is effective in myeloma *in vitro* and *in vivo* [11, 14, 172]. However, treatment of myeloma cells with compounds such as Hsp90 inhibitors (17-AAG, NVP-AUY922), bortezomib and dexamethasone is usually accompanied by the upregulation of other heat shock proteins such as Hsp70 and Hsp27, protecting cells from apoptosis and contributing to drug resistance [14, 173, 174]. For instance, it has been shown that Hsp27 is overexpressed in myeloma and inhibits the release of Smac, an activator of caspases from the mitochondria, thereby confering dexamethasone resistance [175]. Blocking the Hsp27 upregulation associated with bortezomib treatment overcomes bortezomib resistance [176], and inhibiting p38 MAPK, an upstream regulator of Hsp27, sensitises myeloma cells to bortezomib induced cell death by downregulating Hsp27 [177]. Evidence for HSP90 inhibition having a cytostatic effect on colon adenocarcinoma cells, rather than inducing cell death, suggests that HSP90 inhibitors used in combination with other agents can enhance tumour cell kill [178]. Further studies in myeloma models will be needed to explore possible drug combinations with HSP90 inhibitors.

The cytoplasmic Hsp70s, inducible Hsp72 and constitutively expressed Hsc70 are frequently overexpressed in myeloma [179]. Inhibition of Hsp70 is also effective in inducing myeloma cell death both *in vitro* and *in vivo* [179-181]. As they function as co-chaperones of Hsp90, inhibition leads to Hsp90 client protein depletion as well as cell death [179, 180]. Inhibition of Hsp90 typically leads to a compensatory upregulation of Hsp72, and inhibiting both Hsp70 and Hsp90 results in a more effective anti-tumour response than inhibiting Hsp90 alone [179, 180, 182]. It has also been shown that Hsc70 and Hsp72, sharing 85% sequence homology, have compensatory yet distinctive roles in immunoglobulin folding and survival of myeloma [180, 182].

As Hsf1 is a major transcription factor responsible for the rapid induction of heat shock proteins during stress, recent studies have also explored its role as a potential therapeutic target. Hsf1 regulates gene expression of heat shock proteins such as Hsp90, Hsp72 and Hsp27, as well as non-chaperone genes potentially utilized by cancers such as the tumour necrosis factor (TNF) receptor [104]. High level of Hsf1 is associated with cancer malignancy and poor prognosis clinically, and there is considerable evidence for the direct involvement of Hsf1 in tumourigenesis in cancers including myeloma [183, 184]. Early studies also suggest that inhibition of Hsf1 induces apoptosis in myeloma cells *in vitro* and reduces tumour growth *in vivo*, and this is associated with lowered expression of multiple downstream heat shock proteins.

### Development of heat shock protein inhibitors for the treatment of multiple myeloma

The activity of various inhibitors of the heat shock response is currently being explored, however to date only inhibitors of Hsp90 have reached the advanced stages of clinical development.

### Hsp90 inhibitors

The development of Hsp90 inhibitors was initially based on the natural product geldanamycin, which has potent anti-tumour activity in a wide range of tumour cell lines. Geldanamycin binds to the N-terminal domain of Hsp90, blocking the site of ATP binding and hydrolysis [185]. A number of geldanamycin derivatives have since been developed with improved solubility, stability and toxicology [186]. The geldanamycin based tanespimycin (17-AAG) was the first to enter the clinic as it showed single agent activity *in vitro* on myeloma cell lines [12, 13], and combination treatment with bortezomib led to an increased accumulation of ubiquitinated proteins compared to single agent exposure [187]. Despite encouraging initial results, the development of tanespimycin has since been discontinued. Retaspimycin (IPI-504) is a derivative of 17-AAG thought to be more potent and less toxic to the liver than 17-AAG. A phase I trial showed that it is well tolerated in myeloma patients [188], with similar synergy when combined with bortezomib [189].

In addition to geldanamycin based compounds, a number of novel Hsp90 inhibitors have recently been developed and are undergoing preclinical and clinical studies. NVP-AUY922 (VER52296) efficiently induced apoptosis in myeloma cells at nanomolar concentrations and triggered changes in the molecular signature of cells characteristic of Hsp90 inhibition [14]. Phase I/II studies of AUY922 with and without bortezomib, with or without dexamethasone are currently being performed in patients with relapsed or refractory multiple myeloma (NCT00708292). KW-2478 is another promising novel compound discovered through a unique lead optimization strategy including microbial screening, X-ray crystallography, cell-based screening and *in vivo* models [15]. A study on myeloma cell lines showed that KW-2478, a novel non-purine analogue antagonist, induced growth inhibition and apoptosis associated with Hsp90 client protein depletion [15], and combination with bortezomib exhibited synergistic activity *in vitro* and *in vivo* [190]. Phase I/II Study of KW-2478 in combination with bortezomib in multiple myeloma is ongoing (NCT01063907).

Two orally available Hsp90 inhibitors, NVP-HSP990 and PF-04929113 (SNX5422) have also been tested in myeloma. Preclinical studies show that NVP-HSP990 has potent activity against myeloma and is synergistic with melphalan, histone deacetylase (HDAC) inhibitors and a PI3-kinase/mTOR inhibitor, providing a rationale for early clinical trials [191, 192]. A phase I trial on PF-04929113, a highly selective small molecule Hsp90 inhibitor, has shown encouraging responses in patients with refractory myeloma. Phase II studies are currently being considered [193].

PU-H71, is an emerging purine scaffold HSP90 inhibitor that can not only bind a larger scope of HSP90 conformations compared to its geldanamycin-derived predecessors, but is also unaffected by HSP90 phosphosphorylation. PU-H71 exhibits potent anti-myeloma activity in cell lines by inhibiting both the cytoplasmic and ER resident Hsp90 (Grp94) resulting in the activation of the UPR and caspase dependent apoptosis in myeloma cell lines [194] [195].

### Hsp70 inhibitors

The consistent upregulation of Hsp70s following Hsp90 and proteasome inhibition, and their proven anti-apoptotic roles contributing to drug resistance leads to a growing interest in the development of Hsp70 inhibitors to be used as single anti-cancer agents or in combination with conventional or targeted chemotherapies. However, to date few Hsp70 specific inhibitors have been identified. Two Hsp70 specific compounds, Ver-155008 and MAL3-101, have been tested on myeloma in the preclinical setting.

Ver-155008 is an ATP-analogue capable of inducing caspase dependent apoptosis in a panel of myeloma cell lines via the modulation of multiple oncogenic pathways and enhancing Hsp90 inhibition induced cell death [179, 180]. In contrast to Ver-155008, MAL3-101 inhibits the ability of Hsp40 cochaperone to stimulate Hsp70 ATPase activity, thereby blocking Hsp70 functions in cells [196]. MAL3-101 exhibited promising anti-myeloma properties on myeloma cell lines *in vitro* and *in vivo*, and demonstrated synergy with proteasome and Hsp90 inhibitors [181]. Although these compounds have limited potency, they may form the basis for the development of future derivatives suitable for the clinical setting [197].

### Hsf1 inhibitors

As an alternative to targeting individual heat shock proteins, there has been an interest in the development of inhibitors against Hsf1, the ‘master regulator’ of heat shock response. Since the inhibition of a single heat shock protein such as Hsp90 inevitably leads to the compensatory upregulation of other heat shock proteins such as Hsp70 and Hsp27, targeting Hsf1 instead of the individual chaperones separately is potentially more therapeutically effective, as inhibition of Hsf1 could in theory abolish the ability of a cancer cell to activate the whole heat shock response during cellular stress. The increased sensitivity of hepatocellular carcinoma and melanoma cell lines to HSP90 inhibition with HSF1 knocked down *in vitro*, illustrates the therapeutic potential of an HSF1 inhibitor in combination with HSP90 inhibition [198]. While several small molecular compounds can interfere with the transcriptional activation of Hsf1 or the downstream translational mechanisms, the precise mechanisms of how these compounds work remains unclear and hence, they are not yet valid for clinical investigation [199]. As the development of inhibitors against transcriptional factors lacking obvious druggable sites is challenging, a better understanding of the molecular mechanism controlling Hsf1 activation and function will aid the development of specific inhibitors against this transcription factor [200].

## CONCLUSION

It is becoming increasingly apparent that targeting individual cellular stress pathways or components may not be sufficient for killing myeloma cells as other compensatory pathways or components can be upregulated. Therefore, targeting multiple oncogenic and signalling pathways simultaneously may be the future of myeloma treatment, and cancer treatment in general.

The fact that cancers such as myeloma rely on the protein handling pathway for survival creates a ‘therapeutic window’ for heat shock protein inhibition. Evidence suggests that the inhibition of heat shock proteins affect cancer cells more than normal cells [180, 182], making them attractive as potential therapeutic targets in cancer and encouraging results are observed in the early clinical trials on Hsp90 inhibitors. As individual protein families, heat shock proteins are capable of supporting multiple pathways critical to myeloma survival and progression and inhibiting individual heat shock proteins lead to myeloma cell death. The cell death effect can also be significantly enhanced by combining heat shock protein inhibition with inhibitors of other protein handling pathways, such as proteasome and HDAC inhibitors. Targeting multiple heat shock proteins at the same time can also be a good strategy, exemplified by the enhanced cell killing following dual inhibition of Hsp90 and Hsp70.

Challenges however remain in the effective targeting of these proteins in myeloma. Firstly, the molecular mechanisms of heat shock proteins are still not fully understood, with multiple isoforms of the same heat shock protein playing distinct or compensatory roles. This is exemplified by the consistent upregulation of Hsp72 following Hsc70 inhibition, and inhibition of both isoforms may be required. Understanding the roles of individual heat shock proteins and the effect of combined inhibition of multiple heats shock proteins is the key to developing an effective treatment strategy against myeloma with minimal side effects in patients. Secondly, the development of heat shock protein inhibitors suitable for the clinic remains a major challenge. Apart from Hsp90 inhibitors, inhibitors against Hsp70, Hsp27 and Hsf1 are still in the early phase of development despite strong evidence of their involvement in myeloma survival. Thirdly, preliminary data shows enhanced myeloma cell killing by combining heat shock protein inhibition with inhibition of other pathways such as the proteasome, but the best combination treatment strategies are yet to be established.

In conclusion, targeting the heat shock pathway is a promising therapeutic strategy in myeloma as well as in other cancers. Much work is currently ongoing in this area and the results are eagerly awaited.
